# Fellow-Workers and the Roll of Honour

**Published:** 1915-09-25

**Authors:** 


### ROUND THE WORLD.

[The Editor invites Early Information and Contributions Marked "Fellow-Workers."]

### Fellow-Workers and the Roll of Honour.

THE steadily increasing list of medical students killed as combatants includes the names of Second-Lieutenant R. J. T. Ingram-Johnson, the elder son of Dr. R. E. Ingram-Johnson, of Southmoor, Durham; Second-Lieutenant W. A. Stanwell, formerly at Guy's, the only son of Mr. W. Stanwell, M.R.C.S., of Rochdale; Lance-Corporal George Auchinachie (Gordon Highlanders), who, previously to the war, had been studying at Aberdeen University; Lieutenant Richard Patrick Tobin, a student of Trinity College, Dublin, and son of a surgeon in that city; Captain John Theodore Spencer Weston, of Birmingham University, who had already won the Military Cross; and Lieutenant John MacAdam, formerly of St. Bartholomew's Hospital.

IT is indicative of the responsible position now given to scientific medicine by the State that of the eleven members of the committee appointed to consider questions affecting the well-being of workers in munition factories, six are medical men occupying influential places in their profession. These gentlemen are: Sir George Newman, Sir Thomas Barlow, Bart., Dr. E. L. Collis, Dr. W. M. Fletcher, Dr. Leonard E. Hill, and Professor A. E. Boycott.

SIR GEORGE NEWMAN, M.D., C.M. Edin., D.P.H., F.R.S.E., who will act as chairman of the new committee, is chief medical officer to the Board of Education and lecturer on public health to St. Bartholomew's Hospital. One of our foremost authorities on all public health matters, he has written extensively on his special subject.

A PAST president of the Royal College of Physicians, consulting physician to University College Hospital and to Great Ormond Street Hospital for Sick Children, and the possessor of innumerable academic honours and distinctions, Sir Thomas Barlow, M.D., F.R.S., suitably represents general medicine on the committee in question. He was formerly physician extraordinary to Queen Victoria and physician to the Royal Household.

DR. EDGAR LEIGH COLLIS is Inspector of Factories for the Home Office and a recognised authority on all matters pertaining to the health of workers in factories and workshops. A student of St. Thomas's Hospital, he qualified M.R.C.S., L.R.C.P in 1896, becoming an M.B., B.Ch. Oxon. in the following year. Dr. Collis delivered the Milroy Lectures of the Royal College of Physicians this year.

DR. WALTER MORLEY FLETCHER, M.D. Cantab., D.Sc., F.R.S., a former "Bart.'s" student and colleague of the above, is secretary of the Medical Research Committee constituted under the National Health Insurance Act. A physiologist who has published important original observations, he was at one time senior demonstrator of physiology at Cambridge.

DR. LEONARD ERSKINE HILL, M.B. Lond., F.R.S., is well known in hospital circles as the brilliant physiologist who for some years lectured at the London Hospital Medical College. An investigator whose work is known in scientific societies throughout the world, Dr. Hill is now director of the department of applied physiology and hygiene organised under the aforementioned Medical Research Committee.

PROFESSOR ARTHUR EDWIN BOYCOTT, M.D. Oxon., F.R.S., formerly lecturer on pathology at Guy's Hospital, now occupies the chair of pathology in Manchester University. A St. Thomas's man, he graduated in medicine and surgery in 1902. He also has carried out many important researches in physiology and pathology.

SURGEON DAVID REVELL BEDELL SIVRIGHT, R.N., who died in the Near East early this month, was at one time a famous exponent of Rugby football, and was well known as a Scottish "international." A student of both Edinburgh and Cambridge Universities, he graduated in medicine and surgery at the former in 1910, and after holding resident appointments at the Edinburgh Royal Infirmary settled in private practice. In January of this year Mr. Sivright entered the Royal Navy as a temporary surgeon, and became attached to the Field Ambulance of the Royal Naval Division. It is to be noted that he also excelled in fields of sport other than football, for a few years ago he held the championship in Scotland for amateur heavy-weight boxing.

THE Princess Christian British Red Cross Hospital, near Egham, which has scarcely taken three months to build, is under the direction of Sir William Taylor, K.C.B., as commandant, and Major Buckley, R.A.M.C., as assistant commandant. Sir William Taylor, who took the M.D., C.M. degrees of Glasgow University in 1864, is an honorary physician to His Majesty the King, and retired from the Army Medical Service with the rank of Surgeon-General after a distinguished career therein.

IT can no longer be doubted that in the sinking of the *Royal Edward* four gallant medical officers lost their lives—namely, Lieutenant-Colonel J. H. Dauber, Major J. Mowat, Captain C. B. Marshall, and Lieutenant T. Hayhurst.

LIEUTENANT-COLONEL JOHN HENRY DAUBER, R.A.M.C. (T.F.), was a West End practitioner, who had for a good many years been connected with the Territorial branch of the R.A.M.C. Always interested in gynæcology, he was at one time registrar to the Hospital for Women, and had published several papers on this special subject. Qualifying in 1889, Lieutenant-Colonel Dauber later added to his name the letters F.R.C.S.I., M.B., B.Ch. Oxon., M.R.C.P. Lond.

CAPTAIN CHARLES BERTRAM MARSHALL, R.A.M.C. (T.F.), was a Manchester student, who took the M.B., Ch.B. in 1909. Subsequently proceeding to the M.D. degree and taking the D.P.H., he held several resident posts in Manchester, and eventually settled in practice in Cheshire. Soon after the outbreak of war he took a commission, and was posted to the 3rd East Lancashire Field Ambulance.

LIEUTENANT THOMAS HAYHURST, R.A.M.C. (T.F.), had only been qualified about four years. An Edinburgh man, he spent some time after graduating in residence at the Victoria Hospital, Burnley, before starting in private practice. Last autumn he joined the 1st East Lancashire Field Ambulance.

